# Understanding the impact of antibiotic perturbation on the human microbiome

**DOI:** 10.1186/s13073-020-00782-x

**Published:** 2020-09-28

**Authors:** D. J. Schwartz, A. E. Langdon, G. Dantas

**Affiliations:** 1 Department of Pediatrics, Division of Infectious Diseases, Washington University School of Medicine in St. Louis, St. Louis, MO 63110 USA; 2 The Edison Family Center for Genome Sciences & Systems Biology, Washington University School of Medicine in St. Louis, St. Louis, MO 63110 USA; 3 Department of Pathology and Immunology, Division of Laboratory and Genomic Medicine, Washington University School of Medicine in St. Louis, St. Louis, MO 63110 USA; 4 Department of Biomedical Engineering, Washington University in St. Louis, St. Louis, MO 63110 USA; 5 Department of Molecular Microbiology, Washington University School of Medicine in St. Louis, St. Louis, MO 63110 USA

**Keywords:** Gut microbiome, Resistome, Antibiotics, Perturbation, Resilience, Dynamics, Recolonization

## Abstract

The human gut microbiome is a dynamic collection of bacteria, archaea, fungi, and viruses that performs essential functions for immune development, pathogen colonization resistance, and food metabolism. Perturbation of the gut microbiome’s ecological balance, commonly by antibiotics, can cause and exacerbate diseases. To predict and successfully rescue such perturbations, first, we must understand the underlying taxonomic and functional dynamics of the microbiome as it changes throughout infancy, childhood, and adulthood. We offer an overview of the healthy gut bacterial architecture over these life stages and comment on vulnerability to short and long courses of antibiotics. Second, the resilience of the microbiome after antibiotic perturbation depends on key characteristics, such as the nature, timing, duration, and spectrum of a course of antibiotics, as well as microbiome modulatory factors such as age, travel, underlying illness, antibiotic resistance pattern, and diet. In this review, we discuss acute and chronic antibiotic perturbations to the microbiome and resistome in the context of microbiome stability and dynamics. We specifically discuss key taxonomic and resistance gene changes that accompany antibiotic treatment of neonates, children, and adults. Restoration of a healthy gut microbial ecosystem after routine antibiotics will require rationally managed exposure to specific antibiotics and microbes. To that end, we review the use of fecal microbiota transplantation and probiotics to direct recolonization of the gut ecosystem. We conclude with our perspectives on how best to assess, predict, and aid recovery of the microbiome after antibiotic perturbation.

## Introduction

The human gut microbiome consists of bacteria, viruses, and fungi ideally living symbiotically with their human host, though this review will focus exclusively on bacterial residents within the gut microbiome [1]. Individual species and collective bacterial functions within the gut microbiome confer many benefits throughout life including metabolizing dietary contributions, educating the immune system, defending against pathogens, and contributing to overall health and optimal growth [2–6]. The gut microbiome is affected by and influences pathologies including inflammatory bowel disease (IBD), allergies, asthma, and neurobehavioral disorders [4, 5, 7, 8]. Another key feature of the microbiome is the quantity, identity, and function of antibiotic resistance genes (ARGs), collectively called the resistome. ARGs transmit between species within the gut microbiome including potential pathogens. Therefore, understanding how the resistome changes in parallel with the microbiome is vitally important [9, 10]. Accordingly, numerous avenues of research are being pursued to understand what constitutes healthy and abnormal microbiomes and resistomes.

Current microbiome research is largely concerned with “who is there?” and “what are they doing?” Microbiome taxonomic profiling is achieved by culture-dependent molecular or phenotypic typing or culture-independent sequencing of taxonomically informative marker genes or whole metagenomic shotgun sequencing from microbiome samples, within or between individuals [11–14]. Similarly, features and functions of the microbiome can be assessed by gene-level analysis, metabolomics, and assessment of the abundance of gene pathways for microbial metabolic function [15–20]. These analyses are typically conducted in the context of human development throughout life or in connection with clinical outcomes [21]. Measures of diversity within (*α*) and between (*β*) samples can be used to compare microbial communities over time and between disease states (extensively reviewed in [22]). Our ability to attribute disease associations to causality is difficult and requires longitudinal, prospective studies, ideally complemented by mechanistic validation in animals [21, 23]. However, important associations between the human microbiome structure and function with diseases and health nevertheless provide meaningful hypotheses and correlations [7, 21, 24, 25].

**Fig. 1:**
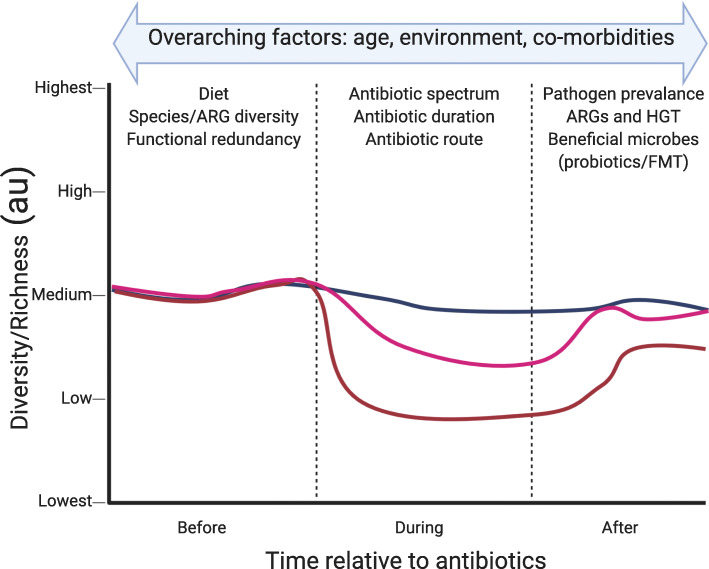
Antibiotic perturbation to the microbiome needs to be considered in context. Certain factors are important to consider throughout life (overarching factors). Other factors such as diet and the functional and species diversity and redundancy are important to consider when the antibiotic perturbation is applied. The duration, spectrum, and route of antibiotics are vitally important in the context of how the microbiome responds during an intervention. The post-antibiotic environment including availability and colonization of pathogens, frequency of horizontal gene transfer (HGT), MDROs, and beneficial microbes is important to consider the resilience and response after antibiotic cessation. These factors influence the structure and function of the microbiome before, during, and after antibiotics throughout life. Created with BioRender

The most common external perturbations to the microbiome are diet, medications (especially antibiotics), and the environment [26–31]. In this review, we focus on antibiotic perturbation throughout life and associations with other factors including age and maturity of the microbiome, diet and the environment, and the co-morbidities of the individual (Fig. 1). How the microbiome responds to antibiotics is altered by the state of the microbiome at the time of perturbation (diet, species, and functional diversity and redundancy) and the strength of the perturbation (route, spectrum, and duration of antibiotics). After cessation of antibiotics, the prevalence of beneficial or potentially pathogenic and/or antibiotic-resistant (AR) microbes that recolonize the gut microbiome governs the initial and long-term outcomes of antibiotic treatment (Fig. 1). These factors must be considered individually and collectively when correlating changes in the microbiome structure and function to human health and disease.

## Development of the human microbiome

Immediately after birth, bacteria, viruses, and fungi colonize the sterile human gut with a subsequent rapid accumulation of species and diversity from the mother and the environment [32–34]. Gut microbiome richness and diversity continue to change until 2–3 years of life, after which the microbiome stabilizes with less dramatic monthly changes [26, 35]. The neonatal and infant periods, however, are defined by dynamic changes in microbial diversity on a weekly, if not daily, timescale. Dramatic strain-level changes and interactions shape the early life microbiome providing essential metabolic and immune regulatory functions [36, 37]. This increase in microbial species and functions matures the gut microbiome increasing both its richness and diversity [26, 38]. Determining geographical and environmental developmental baselines and trajectories allows for understanding the effects of perturbations to normal development [32, 39]. Preterm birth, residence in the neonatal intensive care unit (NICU), malnutrition, and frequent antibiotics can perturb microbiome development and result in microbiota “age regression” [32, 38, 39]. Microbiota age regression is indicated by the child’s chronological age exceeding their age predicted from the constituents of their microbiome [32, 39]. Thus, comparing a child’s chronological age with an assessment of microbiota health from the species composition can provide a gross understanding of development and disruptions thereof.

The overall diversity and community architecture of healthy adult microbiomes do not change dramatically in the absence of significant perturbation [26, 40, 41]. However, among healthy adults, the relative abundance of specific taxa can change on a daily or weekly scale [42], but microbial functions are more stable longitudinally [43]. As an important caveat, some of these inferred differences regarding daily changes may be an erroneous result of sequencing error from various sources and amplification of or depletion of rare and low abundance taxa (Table 1) [44, 48]. Biases can be both taxa-intrinsic as well as protocol-specific [44]. For example, among 3 different extraction protocols, the relative abundance of *Clostridioides difficile* was universally lower and *Fusobacterium nucleatum* higher than the actual abundance of the mock community [44]. Conversely, certain protocols enriched for specific taxa over others in all mock communities. The range of individual abundances among replicates varied as much as 10-fold within a given extraction and processing protocol [44]. Therefore, a daily change in relative abundance could be a result of systematic bias and should be evaluated as such (Table 1).

**Table 1:** Microbiome methods and limitations

Bias introduced during extraction, amplification, sequencing, and bioinformatic processing can alter the relative abundances of species within a sample [44]. Relative abundances can range from 50-fold higher or lower than actual depending on the specific species contribution and protocols used [44]. The complete absence of a species may reflect bias below the limit of detection. Conversely, expansion of specific taxa may reflect progressive and systemic bias enriching for sequencing reads from those taxa [44]. It is therefore important to consider and correct for these biases in any experiment where taxon relative abundance is considered using computational methods [44]. A key step in any metagenomic sequencing experiment is to sequence similar, defined communities of different taxon proportions to understand bias in each protocol. Sequencing-defined communities can lead to computational estimates of protocol bias that can be applied to all samples prior to analysis [44]. Furthermore, extraction and processing introduce contamination depending on its format, and each kit has its own DNA that needs to be evaluated especially when considering a potentially sterile site [45–47]. Thus, sequencing both mock, negative controls of the sequencing kit only and contrived, defined bacterial communities is essential for optimal microbiome sequencing determination.	

Although the dichotomy of stable (e.g., healthy adult) versus dynamic (e.g., developing infant) microbiomes is likely oversimplified, it is important to understand that the impact of an intervention/perturbation depends on the context (Fig. 1). That is, a relatively more stable, healthy, adult microbiome can resist and rebound faster and more completely from the same perturbation that could change the developmental trajectory of a preterm neonate and leave lasting changes (Fig. 1) [32]. Partly, this difference relates to the degree of functional diversity and redundancy in the microbiome with different strains of the same species contributing functions or occupying various distinct and overlapping niches [37, 49].

## Acute perturbations to the microbiome and resistome

### Vulnerable infancy

Large-scale studies of the microbiome have demonstrated that the dynamic first 2 years of life respond most dramatically to antibiotic perturbation [26, 50]. Antibiotic treatment during the first 18 months of life results in greater disruption than subsequent administration, as measured by *β* diversity between consecutive samples [50, 51]. Among the most dynamic periods of microbiome development is the first 6 months of life [26, 28]. Prior to birth, intrapartum antibiotic administration to mothers significantly affected the microbiome structure of 1-month-old neonates relative to control infants even in the absence of continued antibiotic exposure [52, 53] (Table 2). Further, intrapartum antibiotics lead to persistent enrichment of ARGs in exposed, term neonates relative to non-treated neonates or their mothers at 6 months of life [52]. Importantly, in this study, neither exposed nor the control children were antibiotic-treated after birth. Therefore, the administration of antibiotics during this critical developmental window can lead to short- and intermediate-term negative effects on the microbiome and resistome.

**Table 2:** Key findings of summarized work

Authors	Population	General findings	Species and ARGs implicated
Parnanen et al. 2018 [52]	Fecal samples of 16 mother-infant pairs shotgun metagenomic sequenced over the first 6 months of life	Intrapartum antibiotics increased fetal ARGs and decreased diversity at 1 month	Efflux pumps and other ARGs mapping to *E. coli* and *Klebsiella* spp. enriched in antibiotic-exposed subjects
Gibson et al. 2016 [54]	84 NICU-hospitalized preterm neonates with stool samples flanking antibiotic treatment sequenced	Meropenem, cefotaxime, and ticarcillin-clavulanate decreased microbiome diversity whereas gentamicin and vancomycin had variable effects	Abundance of *E. coli* and *S. aureus* and the two-component regulator system, *cpxR/cpxA* predicted gut microbiome response to vancomycin and gentamicin
Bokulich et al. 2016 [55]	43 infants followed over the first 2 years of life	Antibiotics delayed microbiome maturation with fewer species and lower diversity that resolved after 1 year of life	Relative abundance of *Clostridiales* and *Ruminococcus* decreased from 3 to 9 months in the antibiotic-exposed group
Palleja et al. 2018 [56]	12 healthy adults treated with 4 days of meropenem, gentamicin, and vancomycin with fecal shotgun metagenomic sequencing for 6 months after	Gut microbiome diversity recovered after 6 months, but richness did not; no persistent enrichment of ARGs	Multi-drug efflux pumps most enriched immediately after treatment; complete absence at 6 months of baseline species belonging to *Bifidobacterium*, *Coprococcus*, and *Methanobrevibacter* within individuals
Lloyd-Price et al. 2019 [7]	Multi-omic analysis of 132 children and adults with IBD or controls contributing 2965 specimens	Increased inter-individual variation during IBD flare; multi-omic signatures differentiate dysbiosis from baseline	*Prevotella copri* maintained high relative abundance in Crohn’s disease patients but fluctuated in its abundance in controls; dysbiosis marked by decreased *Faecalibacterium prausnitzii* and *Roseburia hominis* and increased *E. coli*
Gasparrini et al. 2019 [32]	41 NICU-hospitalized preterm infants variably exposed to antibiotics and 17 antibiotic-naive near-term infants followed through 21 months of life	Preterm infant microbiome exhibited delayed development with recovery by 15 months	Persistent MDRO Enterobacteriales colonization in several infants; model including *Prevotella copri*, E*ubacterium rectale*, *Ruminococcus* spp., and ARGs 96% predictive of whether a fecal sample originated from a preterm, antibiotic-exposed or near-term antibiotic-naive infant
Yassour et al. 2016 [28]	39 Finnish children aged 2 to 36 months contributing monthly stool samples	Frequent antibiotic courses diminished gut microbiome species and strain diversity and enriched for ARGs	Antibiotic treatment more drastically affected the strain-level diversity of *Bacteroides fragilis* than *Bacteroides vulgatus*; relative abundance of many ARGs decreased after cessation; others (CfxA6 beta-lactamase) remained high
Doan et al. [57, 58]	30 children in Niger randomized to placebo or bi-annual azithromycin for 2 years	No dramatic effect on microbiome diversity or relative abundance	Decreased relative abundance of *Campylobacter* spp.; increased macrolide resistance overall and in *S. pneumoniae* at 24 months
Suez et al. 2018 [59]	21 healthy adults treated with 7 days of ciprofloxacin and metronidazole then randomized to probiotics, autologous FMT, and spontaneous recovery	FMT accelerated and probiotics inhibited microbiome structural and functional recovery	Relative abundance of *Enterococcus casseliflavus* and *Blatia producta* inversely correlated with overall microbiome richness

An extreme example of this paradigm of microbiome dynamics is neonates who are born prematurely and reside in NICUs. Antibiotic treatment of premature infants is both routine and extensive [60–62]. Antibiotic treatment decreases gut microbial diversity and enriches for AR potential pathogens [54]. Antibiotics delivered in the NICU are varied, [63] ranging from relatively short-term exposure with narrow-spectrum agents such as ampicillin or cefazolin to long-term exposure with broad-spectrum agents such as 3rd-generation cephalosporins and carbapenems [54, 64]. The preterm gut microbiota of NICU-hospitalized neonates is dominated by *Escherichia coli*, *Klebsiella* spp., *Enterobacter* spp., and *Enterococcus* spp., which are found in the NICU environment, are often multi-drug resistant, and are causes of bacteremia in this population [54, 65, 66] (Table 2). Antibiotic administration in this environment results in a fundamentally altered and extremely ARG-enriched gut microbiome acutely after antibiotics, but the response to each antibiotic differed based on the microbiome composition [54]. The overall microbiome response to gentamicin and vancomycin could be predicted based on the abundance of the species *E. coli* and *Staphylococcus aureus* and the ARGs/bacterial response regulator *cpxA/cpxR* [54]. Additionally, members of the Enterobacteriales harbored hundreds of novel ARGs conferring functional resistance to beta-lactams, tetracyclines, and aminoglycosides [54]. Further research investigating the strain-level diversity and functional evolution over time in the NICU and after discharge is needed to identify the covariation and consequences of specific antibiotic therapy in the context of birth history, diet, and environment for each individual’s microbiome.

Antibiotic treatment in infancy enriches for AR organisms in the stool as determined by selective culturing and DNA sequencing [32, 54]. To more broadly characterize novel and unknown resistance mechanisms, researchers have utilized functional metagenomics, whereby fecal DNA is cloned into plasmids, introduced into *E. coli*, and plated on selective media [32, 54, 67]. These functionally validated ARGs present in the original fecal samples encode for proteins, the majority of which had not been previously ascribed resistance mechanisms in curated databases [54, 68]. Greater than 40% of these ARGs derive from *E. coli*, *Enterobacter* spp., and *Klebsiella* spp., encoding resistance to commonly used antibiotics in the NICU (penicillins and cephalosporins) as well as antibiotics not used in the NICU (tetracyclines and chloramphenicol) [54] (Table 2). A strength of this approach is that it allows the identification of a broad range of ARGs assuming expression in *E. coli*. Accordingly, these novel ARGs are likely still an underestimate of the total resistome. To date, similar methods have been unsuccessful in gram-positive bacteria. Resistance to antimicrobials that exclusively or preferentially target gram positives such as vancomycin, linezolid, and clindamycin would require other methods to identify. Therefore, frequent antibiotic use during times of microbial change acutely disturbs the microbiome and enriches for potential pathogens and ARGs.

### Dynamic childhood

Childhood is a time of immense microbiome dynamics and environmental changes including dietary shifts and introductions [6, 26, 69]. After vulnerable infancy, the microbiome goes through a developmental stage (3–14 months of age) and a transitional stage (15–30 months) before stabilizing to a near-adult like configuration [26]. Arguably, the most significant microbiome perturbation during early childhood is the transition from breastmilk or formula to solid foods [26, 70]. The pre-weaning microbiome is dominated by *Bifidobacterium* spp. and, thereafter, several microbiome states, so-called enterotypes, account for the bulk of gut community structure [26]. Microbial diversity of a breastfed child after weaning increases commensurate with the accumulation and enrichment of strains responsible for dietary metabolism [26, 27, 37]. Transitions between these states accompany chronological aging, likely reflective of differential environmental exposures including antibiotics, infections, and diet [26, 55, 69].

Antibiotic administration during childhood typically consists of short courses of relatively narrow spectrum agents for respiratory tract and oropharyngeal infections [71]. Intermittent postnatal antibiotic exposure was associated with decreased abundance of Clostridiales and *Ruminococcus* even though the overall number of species and diversity was similar between exposed and unexposed groups after 1 year of life (Table 2) [55]. However, if antibiotics are frequent or in the context of underlying gastrointestinal disturbances or inadequate diet, their effects can be magnified [55, 72]. Specifically, short courses of antibiotics were shown to exacerbate dysbiosis from Crohn’s disease [72]. Thus, antibiotics, diet, and environment acutely impact the developmental trajectory and diversity of the gut microbiome of the developing child.

### Stable adulthood

A child’s microbiome achieves an adult-like configuration with less dramatic changes monthly between 2 and 3 years of age [26, 35]. Assuming that the healthy adult microbiome is completely stable is inaccurate as high temporal resolution studies have shown variability of different body sites over time [48]. However, individuals with the greatest Shannon diversity in the gut microbiome had the smallest temporal variability with most taxa stable over long periods of time in the absence of perturbation [41, 48]. These findings have ushered in one of the central dogmas of the microbiome field: microbiomes with higher diversity are more resilient to perturbation. For instance, researchers treated 12 men with 4 days of a cocktail of broad-spectrum antibiotics (meropenem, gentamicin, and vancomycin) and sequenced their gut microbiome over a 6-month period [56] (Table 2). Immediately after treatment, *Klebsiella* spp., *Enterococcus* spp., and *E. coli* increased in abundance, but by 8 days after treatment, no significant differences were observed in these species, suggesting acute bacterial blooms are short-lived after cessation of antibiotics in this population. Microbial richness and diversity dramatically decreased, but Shannon diversity progressively recovered in the subsequent 6 months. Although at the gross community level, the microbiome was restored, the absolute number of species remained significantly lower from baseline samples [56].

Lumping together all antibiotics is an oversimplification as the antibiotic spectrum clearly impacts the response of the gut microbiome (Fig. 1) [73]. Indeed, computational modeling of the short-term impact of various antibiotics demonstrates that ciprofloxacin, a broad-spectrum fluoroquinolone antibiotic, showed more displacement from microbiome equilibrium than amoxicillin, a narrow spectrum β-lactam antibiotic [74]. Certain strains of the same species do not recover, suggesting potentially lasting consequences. Similarly, oral cefprozil (a β-lactam antibiotic) altered strain-level dynamics shifting the dominant strain within each individual [75, 76]. It is likely that some of the individualization of antibiotic effects on strain-level diversification is due to the copy number variation or expression of specific ARGs [76]. Although certain ARGs were significantly increased immediately after antibiotic treatment, reports of lasting ARG changes vary per study likely reflective of the microbiome composition, spectrum, and route of antibiotic administration, co-morbidities, and other factors (Fig. 1) [56, 75, 76]. However, if a stable microbiome is challenged simultaneously by changing the environment such as foreign travel to high infectious burden areas or intensive care admission and antibiotic treatment, the acute microbiome effects can be more dramatic [77–79]. These results demonstrate the myriad acute changes to the microbiome and suggest the remarkable plasticity of the stable adult microbiome to routine perturbation.

Although the adult microbiome is relatively stable in healthy adults, the microbiome dynamically changes during acute inflammation of gastrointestinal illnesses such as IBD [7] (Table 2). In one study, researchers characterized samples as dysbiotic by computing Bray-Curtis dissimilarity between all samples to a reference set of non-IBD controls. Any samples that scored above the 90th percentile from the median sample-sample difference from the reference set were termed dysbiotic. Using a multi-omics approach, they identified that 24% of samples from patients with Crohn’s disease were dysbiotic both metagenomically and metabolomically, far above the expected 10% threshold. Similarly, during dysbiotic shifts, patients had discursions from expected constitution not only of metagenomes and their associated metatranscriptomes and metabolomes, but also in measurements of inflammation [7]. Both concurrent and antibiotic use in the prior 6 months correlated with gut dysbiosis further exacerbating the inter-individual and intra-individual microbiome compositional differences [72, 80]. Thus, acute, antibiotic perturbations to the microbiome have a greater effect on community structure when the microbiome is dynamically changing due to inflammation.

Another potentially more dynamic time of microbiome structure is in the elderly. Indeed, small studies have shown decreased microbiome diversity in the elderly including decreased Firmicutes and increased Proteobacteria, similar to the neonatal microbiome [81–83]. In contrast to younger adults, the microbiome was more variable in its composition when sampled within 3-month intervals [83]. One potential explanation for this finding is the number and diversity of non-antibiotic medications that have been shown to impact the microbiome [31]. More research needs to be conducted on the elderly microbiome in the context of immune senescence, co-morbidities, and infection susceptibility, but the stability of the human microbiome may decrease at either extreme of age.

## Long-term effects of antibiotic perturbation depend on microbiome state

Understanding the long-term effects of specific antibiotics is vital to limiting the negative consequences of AR development and international spread. Despite the overall stable community structure over time, changes in species composition may persist even after short antibiotic perturbations to the stable microbiome [56, 75, 84]. The immediate environment of an antibiotic recipient is an important source of newly introduced microbes [32, 54, 59, 66, 85, 86]. This simple fact deserves a great deal more attention to ensure reliable, positive long-term outcomes after perturbations to the gut microbiome. Longer-term effects are also highly dependent on stage of life and stability of the microbiome (Fig. 1) [26, 28, 32, 50, 87, 88].

### Vulnerable infancy and dynamic childhood

Frequent antibiotic use in the NICU delays preterm neonatal microbiome maturation initially, but the microbiota recovers to a similar taxonomic composition to antibiotic-naive term controls by 15 months of life [32]. Similar microbiome recovery at 4 years of life has been shown for antibiotic treatment in the first year of life [69]. Thus, even in periods of extreme microbiome turnover, from a gross, structural level, the microbiome is still resilient and converges to a similar architecture with antibiotic-naive infants. Although the overall composition of the microbiome was similar, specific species and ARGs present up to 2 years later distinguished them from healthy controls [32] (Table 2). Specifically, multi-drug resistant organism (MDRO) Enterobacteriales (*E. coli*, *Klebsiella* spp., and *Enterobacter* spp.) acquired in the NICU persisted in the gut microbiome up to 1 year after NICU discharge with greater than 99.997% identity between them. Machine learning algorithms classified fecal samples post-NICU discharge deriving from an antibiotic-naive or frequently exposed infant with 96% accuracy [32]. Among the most significant features distinguishing these samples were ARGs including class A β-lactamases as well as functionally validated ARGs to piperacillin and tetracycline and members of the order Clostridiales.

In addition to allowing MDRO potential pathogens to maintain a foothold within the GI tract, frequent antibiotic exposure in this critical microbiome developmental period diminished strain diversity, enriched for ARGs, and conferred a less stable composition [28]. Researchers collected monthly stool samples from 39 children from 2 months to 3 years variant in their antibiotic usage [28] (Table 2). Twenty of these children received between 9 and 15 antibiotic courses mainly for otitis media and respiratory infections. They found that antibiotic-naive children harbored increased strain-level diversity of *Bacteroides fragilis*, a key commensal important for immune education and bacterial tolerance [28, 89]. Harboring more strains of the same species is believed to be a component of a resilient microbiome as well as providing different functions [37, 90, 91]. It is likely possible to achieve microbiome health and resilience to perturbation either by increased strain-level diversity within a species or by maintenance of a diverse array of different species. In both instances, this taxonomic diversity leads to functional diversity. Intriguingly, the authors also speculated that frequent antibiotic courses could lead to multiple colonization attempts by beneficial species (e.g., *Eubacterium rectale*) in lieu of initial colonization and in vivo evolution. That is, because of frequent antibiotic administration, beneficial microbes are not surviving and undergoing in vivo adaptation, potentially inhibiting the microbiome plasticity expected of a healthy, interconnected, adult microbiome [28, 92]. Consecutive samples from the same individual on antibiotics were less similar to each other than antibiotic-naive children. This result is not surprising as antibiotics are expected to acutely diminish diversity. However, these children also exhibited dramatically higher variance in this measurement even when not directly treated with antibiotics. ARGs also increased during antibiotic treatment, not all of which returned to baseline after antibiotic cessation (Table 2) [28]. Frequent antibiotic courses during childhood can thus lead to long-term consequences such as increased microbiome variability, decreased strain-level diversity, and increased MDRO potential pathogens. Thus, although the overall taxonomic structure recovers, specific entities remain in the pediatric microbiome as evidence of prior antibiotic exposure.

The main arguments against the use of antibiotics in vulnerable populations are the loss of diversity and selection for AR pathogens, which is especially worrisome when patients are immunocompromised. Treating the gut microbiota of children with severe acute malnutrition (SAM) therefore constitutes a special case combining all of the factors. In healthy children older than 2 years, some adult-like stability and resilience to antibiotics should be characteristic. Malnourished children however exhibit an age-regressed microbiome which is often lacking taxa of a healthy child or adult [39]. Specifically, the gut microbiome of children with SAM is enriched for Enterobacteriales and deficient in beneficial microbes such as *Dorea* spp. and *Faecalibacterium prausnitzii* relative to healthy children living in the same geographical area [39]. Broad-spectrum antibiotics could further deplete microbial defenses and inhibit the immune system. Indeed, the relative abundance of *Enterobacteriaceae* dramatically increased in children with SAM during treatment with ampicillin/amoxicillin and gentamicin [39]. The increase of potential pathogens on a background of poor microbiome and immune health could be catastrophic leading to invasive AR bacterial infections.

The data to address the long-term concerns of antimicrobial treatment of SAM is accumulating in placebo-controlled randomized trials of mass drug administration. Azithromycin and amoxicillin have been trialed to improve all-cause mortality and SAM in sub-Saharan Africa [93–95]. Researchers analyzed the gut microbiome profiles of 600 preschool children (average age 32 months) randomized to receive a standard course of oral azithromycin or placebo every 6 months for 2 years [57] (Table 2). At the end of the 2-year trial, no statistically significant differences were present between the groups in microbial richness, diversity, or phylum-level taxonomic composition. This corroborates the research above that the gross architecture is not commonly affected by a single course of antibiotics. When delving deeper into the analysis, the authors discovered a reduction in the relative abundance of 35 species including *Campylobacter* spp., which can cause diarrheal illnesses worldwide [57]. As a trade-off for decreased mortality, azithromycin increased phenotypic AR of pneumococcus isolated from the nares from children who completed the trial [58]. Similarly, ARGs encoding resistance to macrolides (of which azithromycin is a member) were enriched in fecal metagenomes from azithromycin-exposed children. No other phenotypic resistance in pneumococcus was significantly different between the treatment groups, and there were no differences in genotypic resistance determinants in whole fecal metagenomes [57, 58] (Table 2).

Amoxicillin is also routinely prescribed for uncomplicated SAM in sub-Saharan Africa; however, similar concerns about the long-term consequences of this practice abound [96]. To address some of these concerns for amoxicillin as described above, researchers determined rates of extended-spectrum beta-lactamase (ESBL) prevalence in a randomized placebo-controlled trial of amoxicillin for SAM in Niger [93, 97]. ESBL-expressing *Enterobacteriaceae* broadly degrade many β-lactam antibiotics, are rising in global prevalence, are common in Asia and sub-Saharan Africa, and are listed by the CDC as serious threats [79, 98, 99]. Amoxicillin prophylaxis increased the percentage of Nigeran children who harbored ESBL *Enterobacteriaceae* and were negative at baseline as determined by selective culturing [97]. This result suggests either endogenous enrichment of below the limit of detection *Enterobacteriaceae*, de novo acquisition of an ESBL *Enterobacteriaceae*, or in vivo horizontal gene transfer (HGT). Additionally, untreated siblings of amoxicillin-treated children were more likely than placebo-controlled siblings to acquire an ESBL *Enterobacteriaceae*. Therefore, the administration of antibiotics can potentially affect the long-term AR of specific genera and can lead to environmental dissemination. However, given the repeated demonstration of their positive effect on childhood health, we support this practice. Further research demonstrating the environmental impact of increased AR in this population needs to be performed.

### Stable adulthood

The relatively stable adult microbiome requires more sensitive measurement techniques to assess long-term perturbations. Once the microbiome has stabilized around age 2–3 [26, 35], minimal differences in microbiome stability have been observed between bi-monthly samples in children treated with various oral antibiotics [50]. Furthermore, researchers treated 66 healthy adults with one of four oral antibiotics and collected salivary and fecal samples 1, 2, 4, and 12 months after exposure [100]. Although both the fecal and salivary microbiomes were acutely disrupted by antibiotics as measured by Bray-Curtis dissimilarity, no differences persisted at 1 year suggesting long-term stability and resilience to perturbation. An alternative explanation, however, is that the microbiome is meta-stable, and antibiotic perturbation may transition the structure to an alternative, yet still stable (meta-stable) composition with similar numbers of species and diversity. Indeed, modeling existing datasets of healthy humans treated with antibiotics supports this assertion [56, 74, 100].

The response to the same antibiotic appears individualized at the taxa level likely reflective of underlying co-morbidities, local environment, or prior antibiotic history commensurate with ARG abundance (Fig. 1) [84, 101]. Additionally, the microbiome is temporally variable in relative abundance even in the absence of perturbation, but these changes were small compared to the impact of ciprofloxacin [84]. Specifically, members of the *Clostridiales*, among the most abundant pre-ciprofloxacin taxa, were absent in all samples after treatment [101]. Advanced sequencing techniques combined with longitudinal sampling will continue to refine our understanding of the long-term consequences and changes to the microbiome from antibiotic and environmental perturbation.

## Therapeutics to ameliorate microbiome and resistome disruption

Antibiotics affect the microbiome and resistome composition, with the degree of perturbation determined by many factors (Fig. 1) [28, 55, 59]. Ultimately, if an individual’s microbiome is distorted beyond correction after antibiotic cessation and treatment of an underlying condition, microbiome repair can be considered [86, 102–106]. The most common diseases for which this has been trialed are *Clostridioides difficile* infection (CDI) and steroid non-responsive colitis [103, 105, 107]. The most common microbiota restoration methods are fecal microbiota transplant (FMT) whereby stools either from the same individual before disruption (auto) or from a healthy donor (allo) are introduced orally or via enema or probiotics. More recent research has investigated whether FMT can more generally be applied to reduce ARG and MDRO burden of resistance-rich microbiomes [108–111]. Multiple case reports have described successful decolonization of MDRO *Klebsiella* spp., *Pseudomonas*, and vancomycin-resistant enterococci with allo-FMT (reviewed in [110]), though no placebo-controlled trials have yet been conducted for this express purpose. Autologous FMT has been proposed as a mechanism to restore a pre-antibiotic microbiome baseline after disruption [59, 106, 112]. Similarly, probiotics, strains of beneficial bacteria consumed during or after the intervention, are thought to mitigate some of the negative consequences of antibiotics on the microbiome [113–115]. Recently, however, probiotics, autologous FMT, and spontaneous recovery were evaluated for their impact on microbiome recovery after antibiotics in healthy volunteers (Table 2) [59]. Twenty-one subjects had their stool microbiome sampled, then they were treated with 7 days of ciprofloxacin and metronidazole and randomized to twice daily probiotics for 1 month, autologous FMT from a pre-antibiotic baseline sample, or no treatment. Antibiotics disrupted gut microbiome richness and diversity and metabolic pathways devoted to sugar, carbon, and amino acid metabolism [59]. Auto-FMT immediately after antibiotics rapidly corrected this dysbiosis with no difference in Bray-Curtis dissimilarity from baseline 1 day after FMT. Spontaneous microbiome recovery occurred 21 days after antibiotic cessation. Surprisingly, probiotic administration (a commercially available 11 species consortium called Bio-25 [116]) delayed and prevented microbiome structural and functional recovery with significant compositional differences present 5 months after antibiotic cessation. The increased relative abundance of *Enterococcus casseliflavus* and *Blautia producta* correlated with maintained low species richness [59]. An important caveat to this study is that the probiotic was administered after antibiotics in lieu of during treatment which is common in clinical practice [114]. This probiotic has also not been evaluated for its prevention of antibiotic-associated diarrhea, and unless the probiotic species are resistant to the administered antibiotics, they would likely be eradicated during antibiotic treatment. Thus, autologous FMT accelerated and probiotic administration prevented microbiome recovery after antibiotic administration. Although probiotic administration is a common practice, these data suggest this practice may exacerbate microbiome recovery and requires further study.

Despite the benefits of FMT detailed above, the introduction of fecal microbes into another individual is not without risk. Recently, two immunocompromised patients became bacteremic with ESBL producing *E. coli* derived from donor FMT, one of whom succumbed to the infection [117]. Accordingly, the FDA has decreed that all investigational FMT products be screened specifically for MDRO, excluding individuals at higher risk for MDRO colonization and rejecting donor stools that contain MDRO [118]. One other intriguing point shown in recent FMT studies is that during the dynamic period of engraftment, new strains, species, and ARGs appear that were not detectable in either the donor or the recipient [86, 105]. The frequency and reproducibility of this effect show that the post-treatment environment remains a major factor in the ultimate composition of the gut microbiome (Fig. 1). The sheer number of recent metagenomically sequenced FMT studies with publicly available data makes this phenomenon an excellent candidate for a meta-analysis. Accordingly, the fate of the microbiome after antibiotic perturbation depends on the environment, resilience, and availability of microbes in the post-disruption period including FMT or probiotics.

## Conclusions and future directions

The state of the microbiome, the duration, route, and spectrum of antibiotic activity, other co-morbidities, diet, and post-antibiotic environment all factor into the expected acute and chronic disruption and resilience from perturbation (Fig. 1) [7, 28, 32, 43, 55, 57, 59, 74]. With the reduction of sequencing costs and technology coupled with computational pipelines, we are well poised to conduct deep and well-controlled studies of the impact of microbiome and resistome changes on human health. The most important issue for the microbiome field in general is progression from understanding correlation to identifying causal molecular mechanisms [23, 119, 120]. We are entering an era of personalized microbiome medicine, whereby medications or therapies can be tailored not only to the human genetic polymorphisms, but also to the specific microbiome constituents [59, 116]. We can envision in the not too distant future, antimicrobials and therapies are prescribed for their direct anti-pathogen benefit while simultaneously limiting collateral damage to the microbiome and resistome [113, 121, 122]. It is naive to assume that such direct agents will not have their own collateral impacts on microbiome composition [122], but hopefully selective agents will be less disruptive overall. The human gut microbiome is intimately linked to human health and disease [7, 21, 123]. Only through carefully considering the impact of interventions on the microbiome, can we better treat diseases and improve human health.

## Data Availability

The data described in this review are published in the cited manuscripts.

## Abbreviations

ARG: Antibiotic resistance-gene
AR: Antibiotic-resistant/resistance
NICU: Neonatal intensive care unit
IBD: Inflammatory bowel disease
ESBL: Extended spectrum beta-lactamase
SAM: Severe acute malnutrition
MDRO: Multi-drug resistant organism
FMT: Fecal microbiota transplant
HGT: Horizontal gene transfer
