# Correction to: Understanding the impact of antibiotic perturbation on the human microbiome

**DOI:** 10.1186/s13073-021-00846-6

**Published:** 2021-02-12

**Authors:** Drew J. Schwartz, Amy E. Langdon, Gautam Dantas

**Affiliations:** 1 Department of Pediatrics, Division of Infectious Diseases, Washington University School of Medicine in St. Louis, St. Louis, MO 63110 USA; 2 The Edison Family Center for Genome Sciences & Systems Biology, Washington University School of Medicine in St. Louis, St. Louis, MO 63110 USA; 3 Department of Pathology and Immunology, Division of Laboratory and Genomic Medicine, Washington University School of Medicine in St. Louis, St. Louis, MO 63110 USA; 4 Department of Biomedical Engineering, Washington University in St. Louis, St. Louis, MO 63110 USA; 5 Department of Molecular Microbiology, Washington University School of Medicine in St. Louis, St. Louis, MO 63110 USA

**Correction to: Genome Med 12, 82 (2020)**

**https://doi.org/10.1186/s13073-020-00782-x**

It was highlighted that the original article [1] contained only the initials of the authors’ first names. This Correction article shows the full author names. The original article has been updated.
